# The levels of anti-HPV16/18 and anti-HPV31/33/35/45/52/58 antibodies among AS04-adjuvanted HPV16/18 vaccinated and non-vaccinated Ugandan girls aged 10–16 years

**DOI:** 10.1186/1750-9378-9-29

**Published:** 2014-09-01

**Authors:** Miriam Nakalembe, Cecily Banura, Proscovia B Namujju, Florence M Mirembe

**Affiliations:** 1 Department of Obstetrics and Gynaecology, Makerere University, Kampala, Uganda; 2 Department of Child Health and Development Center, Makerere University, Kampala, Uganda; 3 Department of Children’s, Adolescent’s and Adult’s Health, National Institute for Health and Welfare, Oulu, Finland; 4 School of Health Sciences, University of Tampere, Tampere, Finland

**Keywords:** Human Papilloma Virus, Bivalent HPV vaccine, Immune response, Uganda, Adolescents

## Abstract

**Background:**

Data on Human Papilloma virus (HPV) vaccine immune response in sub-Saharan Africa is still sparse yet such knowledge is critical for optimal implementation and monitoring of HPV vaccines. Our primary objective was to evaluate levels of anti-HPV-16/18 antibodies and six other ‘high risk’ HPV (hrHPV) types among the vaccinated and unvaccinated Ugandan girls.

**Methods:**

We conducted a cross sectional study among AS04-adjuvanted HPV-16/18 vaccinated and unvaccinated school girls aged 10–16 years in Western Uganda using purposive sampling. The vaccinated girls were at 18 months post vaccination. After consenting and assenting, data was collected using interviewer administered questionnaires for demographics and sexual history. Blood was drawn from which serum samples were analysed by the multiplex HPV serology technology to determine anti-HPV antibody levels to HPV-16/18 and six other hrHPV types (31, 33, 35, 45, 52 and 58). The antibody levels were expressed as Median Fluorescent Intensity (MFI).

A total of 207 vaccinated [mean age 13.1 years (SD 1.5); range 10-16 years] and 197 unvaccinated girls [mean age 13.6 years (SD 1.3); range 10-16 years] participated in the study. Sexual activity was self reported among 14/207 (6.8%) vaccinated and 5/197 (2.5%) unvaccinated girls. The MFI levels for HPV-16 and HPV-18 were 15 and 20 times higher respectively in the vaccinated girls than in the unvaccinated girls. HPV-16 mean MFI level was 4691(SD 1812; 95% CI: 4438-4958) among the vaccinated compared to 218 (SD 685; 95% CI: 190-252) among the unvaccinated girls. For HPV-18 the mean MFI level was 1615 (SD 1326; 95% CI: 1470-1776) among the vaccinated compared to MFI 103 (SD 506; 95% CI: 88 -121) among unvaccinated girls.

In addition antibody levels to non vaccine hrHPV types (31, 33, 35, 45, 52 and 58) were all significantly higher in the vaccinated group than in the unvaccinated group (p<0.01).

**Conclusion:**

The AS04-Adjuvanted HPV-16/18 vaccinated girls showed a higher level of antibodies to HPV-16/18 and other non-vaccine hrHPV types compared to the unvaccinated girls. This may translate into protection against HPV-16/18 and other hrHPV types.

## Background

Cervical cancer is the second most common cancer in women worldwide and the primary cause of cancer related deaths among women in the developing countries [1]. More than 84% of the estimated 528,000 incident cases that occur annually and more than 88% of the 257,000 deaths caused by cervical cancer occur in developing countries [2]. This makes cervical cancer a major cause of morbidity and mortality in the developing world. The substantial difference in incidence and mortality between the developed and developing world [3] is mainly due to lack of established screening programs and early detection interventions [4]. This demonstrates a clear need for alternative cervical cancer prevention interventions.

Human Papilloma Virus (HPV) is the most common sexually transmitted infection and has been recognized as the necessary cause of cervical cancer [5]. In Uganda, HPV infection is common among both HIV negative and positive young women aged below 25 years with prevalence of ‘high risk’ HPV (hrHPV) genotypes ranging from 41.6% - 75.0% and 23.7% - 67.1% respectively according to a recent review [6]. Therefore, prevention of HPV infection is a critical step in reducing the burden of cervical cancer.

The recently developed prophylactic vaccines against HPV infection are more than 90% effective in preventing infection and high-grade pre-cancer lesions associated with HPV types included in the vaccine [7]. Currently, Uganda has endorsed HPV vaccination as one of the strategies for cervical cancer prevention with the second phase of the national vaccination program about to be rolled out by 2015. In comparison to the developed countries, data on the immunogenicity and safety of the vaccine in Sub-Saharan Africa is still sparse [8] despite a number of African countries approving use of the HPV vaccine.

Therefore, a cross sectional study was conducted in Western Uganda to compare levels of antibodies to HPV-16/18 and other hrHPV types among vaccinated and unvaccinated Ugandan girls at month 18 post vaccination.

## Results

### Study population

**Figure 1:**
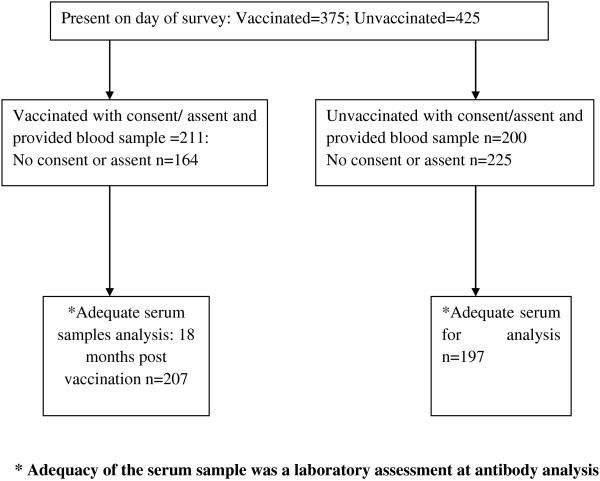
**Flow diagram of vaccinated and unvaccinated girls who participated in the study.** A total of 375 and 425 girls from the vaccinated and unvaccinated districts were present in the schools on the days of the survey. Of those present, 211/375(56.3%) vaccinated and 200/425(47%) unvaccinated girls were recruited in the study. The vaccinated girls were at months 18 post vaccination. In the laboratory, 207/211(98%) and 197/200(98.5%) serum samples from the vaccinated and unvaccinated girls were adequate for analysis respectively.

The results represent two groups, vaccinated and unvaccinated girls. A total of 375 and 425 girls from the vaccinated and unvaccinated districts, respectively, were present in the schools on the days of the survey. Of these, 211/375 (56.3%) and 200/425 (47%) vaccinated and unvaccinated girls assented and had parental consent to participate in the study, respectively (Figure 1). Out of the total 411 vaccinated and unvaccinated girls who consented and assented, 404 (98.2%) had adequate serum samples for antibody analysis. Only girls with adequate serum samples were included in the statistical analysis (vaccinated n = 207: mean age 13.1 years (SD 1.5); range 10–16 years: Non vaccinated n = 197: mean age 13.6 yrs (SD 1.28) range 10–16 years) (Table 1). In both districts, most girls [170/207 (82.2%)] among the vaccinated group and [155/197(78.7)] among the unvaccinated group were 10–14 years old.

**Table 1:** Socio-demographic and sexual characteristics of vaccinated and unvaccinated girls

**Variable**	**Vaccinated n = 207**		**Unvaccinated n = 197**		**P value**
	**n**	**%**	**n**	**%**	
**Age**					**0.001**
**10-14 years**	170	(82.2)	155	(78.7)	
**≥15 years**	37	(17.9)	42	(21.3)	
**School Location**					**0.001**
**Rural**	76	(36.7)	155	(78.7)	
**Urban**	130	(63.3)	42	(21.3)	
**Self reported sexually active**					**0.045**
**Yes**	14	(6.8)	5	(2.5)	
**No**	193	(93.2)	192	(97.5)	
**Age group of sexually active: n = 14 vaccinated; unvaccinated n = 5**					**0.31**
**10-14 years**	11	(78.6)	2	(40)	
**≥15 years**	3	(21.4)	3	(60)	
**Age group of first sexual encounter: n = 16**^**1**^					**0.21**
**10-14 years**	11	(100)	4	(75)	
**≥15 years**	0	(0)	1	(25)	

Socio demographic and sexual characteristics of vaccinated and unvaccinated girls. Mean age for vaccinated and unvaccinated girls was 13.1 yrs (SD 1.5; range 10–16 years) and 13.6 yrs (SD 1.3; range 10–16 years) (p <0.001), respectively.

Overall 19/385 (4.9%) girls self reported engaging in penetrative sexual activity. Fourteen girls (14/207; 6.8%) were vaccinated girls while 5/197 (2.5%) were unvaccinated girls. Median age of sexual debut was 11 years (range 6–13); vaccinated 10.9 years while unvaccinated 12.5 years. There was significant difference in sexual activity between the girls who were vaccinated and unvaccinated (p < 0.001).

^1^Data on age of first sexual encounter for 3 girls is missing.

### Sexual activity

Overall 19/404 (4.7%) of the girls self reported engaging in penetrative sexual activity. Among those who self reported engaging in sexual activity, 14/207 (6.8%) were vaccinated while 5/197 (2.7%) were unvaccinated girls. Overall, the median age at sexual debut was 11 yrs; range 6–13 years (vaccinated 10.9 years while unvaccinated 12.5 years; Table 1).

### Immunogenicity

**Figure 2:**
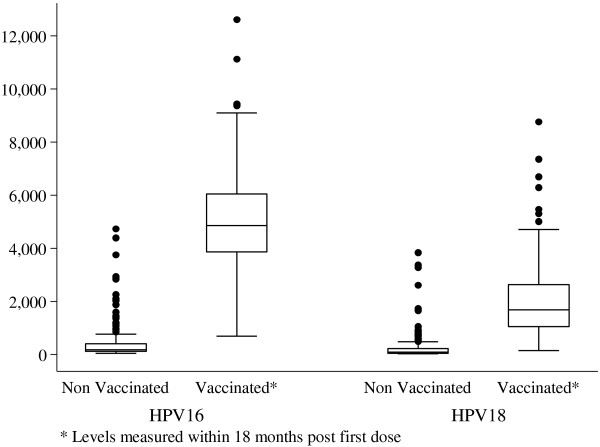
**Comparison of the MFI antibody levels to HPV-16 and HPV-18 at month 18 post vaccination among the vaccinated girls and the unvaccinated girls.** The MFI levels for HPV-16 and HPV-18 among the vaccinated girls were over 20 and 15 times higher respectively among vaccinated than the unvaccinated girls. The central line represents the median MFI levels; above and below the median line in the box are the 75^th^ and 25^th^ centiles; the whiskers represent the upper and lower MFI values and the dots represent the outlier value. The outlier values were calculated using the z –scores (standardizing the data) with an absolute value of z as 1.96. There were 13/207(6.3%) and 11/197(5.5%) vaccinated and unvaccinated girls respectively with outlier values of antibody levels to HPV-16. For HPV-18, 11/207 (5.3%) and 6/197(3%) from the unvaccinated group had outlier values. For both HPV-16/18, the girls who had outlier values were comparable in terms of age and sexual activity.

The MFI level for HPV-16 and HPV-18 at month 18 post vaccination was 15 and 20 times respectively higher among the vaccinated girls than the unvaccinated girls (Figure 2). HPV-16 mean MFI level was 4691 (SD 1812; 95% CI: 4438–4958) among the vaccinated compared to 218 (SD 685; 95% CI: 190–252) among the unvaccinated girls. For HPV-18 mean MFI level was 1615 (SD 1326; 95% CI: 1470–1776) among the vaccinated compared to 103 (SD 506; 95% CI: 88–121) among the unvaccinated girls (Table 2).

**Table 2:** Showing the MFI log10 transformed antibodies to HPV-16/18 and other hrHPV types among vaccinated and unvaccinated girls

	**Vaccinated month 18 (n = 207)**	**Unvaccinated (n = 197)**	
	**MFI (95% CI)**	**MFI (95% CI)**	**P-value**
**HPV16**	4691.4(4438.1-4957.9)	218.5(189.7-251.6)	<0.001
**HPV18**	1615.5(1469.6-1775.8)	103.3(88.1-121.3)	<0.001
**HPV31**	844.9(750.1-951.5)	251(211.3-298.3)	<0.001
**HPV33**	444(399.3-493.6)	245.7(215.2-280.5)	<0.001
**HPV35**	752(661.3-854.9)	185.2(158.7-216.1)	<0.001
**HPV45**	634.6(563.2-715.2)	127.6(111.7-145.7)	<0.001
**HPV52**	640.2(570.4-718.5)	260.6(231–294)	<0.001
**HPV58**	404.9(364.6-449.5)	229.1(207.3-253.3)	<0.001

Comparing means of MFI log_10_ transformed antibodies to HPV-16/18 and other hrHPV types among vaccinated and unvaccinated girls. The mean of the MFI levels to other non vaccine hrHPV-31, 33, 35, 45, 52 and 58 were all significantly higher among the vaccinated than the unvaccinated girls when compared antibodies at month 18 post vaccination.

**Figure 3:**
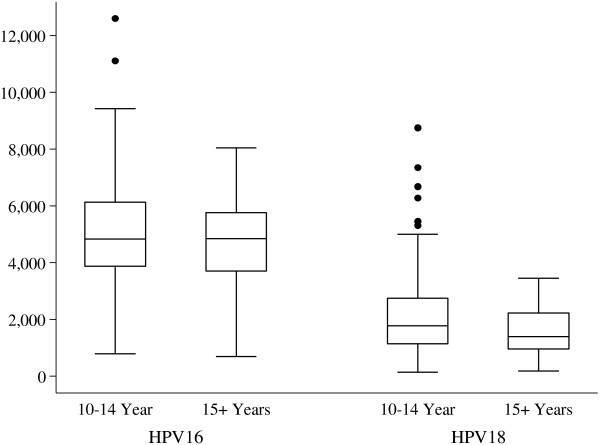
**Comparing the median of antibody levels to anti HPV-16/18 among 10–14 year old and those ≥15 years old vaccinated girls.** The central line represents the median of the MFI levels; above and below the median line in the box are the 75^th^ and 25^th^ centiles; the whiskers represent the upper and lower MFI values and the dots represent the outlier value

**Figure 4:**
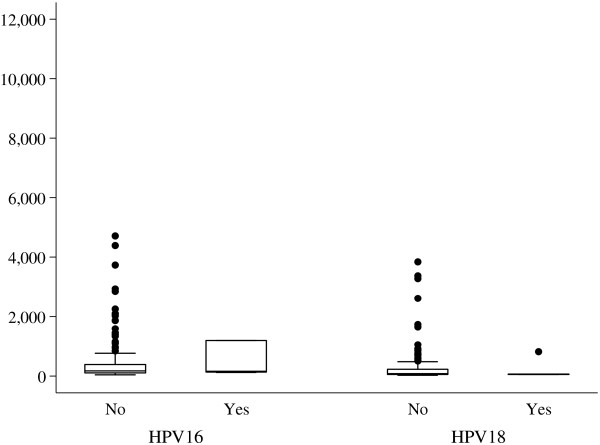
**Comparing the median antibody values to anti HPV-16/18 unvaccinated girls who self reported being sexually active and those who were not.** The central line represents the median of the MFI levels; above and below the median line in the box are the 75^th^ and 25^th^ centiles; the whiskers represent the upper and lower MFI values and the dots represent the outlier value.

There was no difference in the MFI levels for anti HPV-16/18 between vaccinated girls who were 10–14 years and those who were 15 years and older: HPV-16 (*p* =0.47); HPV-18 (*P* = 0.1) (Figure 3). Likewise, among the unvaccinated girls, there was no difference in the MFI levels for anti HPV-16/18 between the self reported sexually active and those who didn’t report. HPV 16 (*p* =0.43); HPV-18 (*P* = 0.58) (Figure 4).

Further, the transformed MFI levels to other non vaccine hrHPV-31, 33,35,45,52 and 58 were all significantly higher among the vaccinated than the unvaccinated girls (Table 2).

## Discussion

This is one of the few studies so far evaluating antibody responses to the bivalent AS04-adjuvanted HPV-16/18 vaccine in Sub-Saharan Africa. We observed a higher level of antibodies to HPV-16/18 and other non vaccine hrHPV types 31, 33, 35, 45, 52, 58 among the vaccinated girls when compared to the unvaccinated ones. By comparing vaccinated girls with unvaccinated girls in this study, we were able to demonstrate that vaccination with the bivalent AS04-adjuvanted HPV-16/18 vaccine may have been responsible for the high antibody levels to the vaccine hrHPV-16/18 and other non vaccine hrHPV types in this population of girls.

The high immune response to HPV-16/18 among vaccinated girls in our study agrees with the results of immunogenicity studies among HIV negative girls from sub-Saharan Africa as well as other parts of the world [8-10]. Although there is not yet published data showing levels of antibodies to HPV-16/18 vaccine using the multiplex serological method, this method still demonstrated HPV-16/18 antibody levels that were many times higher among the vaccinated girls as compared to those observed among the unvaccinated girls. This trend of antibody levels higher than natural infection expressed as geometric mean titres has been demonstrated in one of the longest follow up studies of the bivalent HPV-16/18 vaccine using ELISA by the vaccine manufacturers (GlaxoSmithKline (GSK) Biologicals) [11].

Furthermore, results of this study have showed higher antibody levels to non vaccine hrHPV types 31, 33, 35, 45, 52, 58 among the vaccinated girls as compared to the unvaccinated girls. This observation is in line with what has already been documented in other studies that HPV-16/18 vaccine induces cross-neutralising antibodies that may offer cross protection among phylogenetically related HPV types (α7 (HPV18 and 45) or α9 (HPV16, 31, 33, 52 and 58) [12,13]. Likewise in this study, the observed antibody levels to the non vaccine hrHPV types may translate into cross protection. However, because the immune correlates of protection to all the hrHPV types are yet to be determined, the results from this study may not conclude on the issues of cross protection.

In previous trials, the strong vaccine induced immune response has been proved to protect against persistent HPV-16/18 infections and CIN disease [9,10]. We assume that the much higher immune response observed among these girls compared to their unvaccinated counterparts may translate into protection against HPV infection, CIN and eventually cervical cancer. However it will be many years before this is realised, therefore there is need for close follow up especially in this environment where cervical cancer is very prevalent.

The high level of immune response to the vaccine seen in this study may support the idea of fewer doses in this age group as has been endorsed by the World Health Organisation (WHO) [14] though this study did not investigate the immune response according to different doses. Further, in Africa as elsewhere, the duration of protection as well as the immune correlate of protection is yet to be known. Therefore, as HPV vaccination programs continue to be rolled out in Africa, it is necessary to continue monitoring HPV-16/18 vaccine performance as well as obtaining data on the most cost effective vaccination schedules all over the region.

The girls were at the same school level in the different schools although there was a significant difference in their age and sexual activity. The vaccinated girls were mainly from around the urban area and tended to be younger. This observation is in line with what is already known that Ugandan children from rural settings tend to start school at an older age than their urban counterparts [15]. Further, our study shows evidence of sexual activity below the Uganda national mean age of sexual debut which is 17.5 years. However, this national age of sexual debut is from a larger sample size stretching over a wider age range as compared to the girls who participated in our study. The finding of sexual activity in this age group is consistent with results of other studies which showed that in Uganda and other sub-Saharan countries 6%-11.1% of girls in the age range of 10–15 years had already initiated sex [16,17]. Though the girls from the vaccinated district tended to be younger, they were more likely to have engaged in sex as compared to the unvaccinated girls. The level of self reported sexual activity observed among the unvaccinated girls in our study agrees with what has been documented in a recent publication [18] where 2.5% of the unvaccinated girls from another district volunteered information that they were sexually active. However unlike the results of our study, this publication did not find a difference in sexual behaviour among the girls who were vaccinated and those who were not. The difference in this finding between the two studies may be due to the different cultural settings as well as location of the girls in terms of urban and rural despite the girls being of a similar age group and HPV vaccination status.

Among the unvaccinated group, when sexually active girls were compared with those who were not, there was no difference in MFI levels to both HPV-16/18. The sexually active girls may not have been exposed to HPV-16/18 despite the sexual activity. Further, it is known that about 50% of those exposed to HPV infection may not mount an immune response [19] which may explain the lack of difference in MFI levels. In addition, our study had few numbers that self reported sexual activity. Finally, self reporting sexual activity may not be a very reliable way of assessing this factor as has been documented in other studies [20,21].

Association between sexual activity and levels of antibodies to HPV-16/18 was not assessed for the vaccinated girls as results from some studies [8,22] so far have showed that natural exposure to HPV-16/18 may not influence immune response to the HPV-16/18 vaccine. Further, evidence [8,23] shows that girls in the age range of 10–14 years had higher antibody titres to the HPV vaccine than those 15 years and above an observation that was not evident in our study. However, in the studies above, the age span of the study participants was longer (15–25 years) with more numbers whereas in our study the age span was only 15–16 years with fewer girls. The difference in the age ranges (15–16 versus 15–25 years) together with the fewer numbers in our study may explain why we have not seen a difference in vaccine induced antibody levels as far as age is concerned.

This study had some limitations. The antibody levels were only assessed at one time point. There was no baseline antibody level from the vaccinated girls before vaccination. However for a similar picture of levels of anti-HPV16/18 after natural exposure to HPV-16/18 a group of girls from a neighboring district who would have been vaccinated at the same time were chosen to participate in this study as a comparative group. This inclusion of the unvaccinated group of girls also enabled a demonstration of a difference in antibody levels which could only be explained by vaccination. The girls were of unknown HIV status. This is the situation that will happen in normal vaccination programs; HIV status will not be exclusion for the HPV vaccine. The method used for HPV serological analysis is not yet widely used to monitor HPV vaccine immunogenicity and comparison with other studies may be difficult, however comparison by trends of antibody levels may be appropriate for future follow up even when different methods are used.

## Conclusion

The AS04-adjuvanted HPV-16/18 vaccinated girls showed a higher level of antibodies to HPV-16/18 and other non-vaccine hrHPV types compared to the unvaccinated girls.

These results support continued use of the prophylactic HPV-16/18 vaccine in Africa where for decades there has been no success with any screening program against cervical cancer, an intervention that has helped to significantly reduce cervical cancer in the developed world.

Therefore for African countries, effective HPV vaccination programs seem to be the window of opportunity to reduce cervical cancer mortality and morbidity. By assessing both vaccinated and unvaccinated girls, we are able to provide insight in the immunological response to the HPV-16/18 vaccine as well as provide baseline knowledge of HPV-16/18 and other hrHPV types after natural exposure. It is also evident from this study that some girls initiate sex earlier than expected therefore efforts to vaccinate girls at the earliest WHO recommended age of 9 years [14] should be promoted. The knowledge generated from this study is important for future vaccine monitoring and implementation. Follow up studies to assess the immunogenicity of the vaccine over time are recommended since it will be many years before the effect of the vaccine is realized [24].

## Materials and methods

### Study design

We conducted a cross sectional survey between October 2011-February 2012 among HPV vaccinated and unvaccinated girls at 18 months post vaccination. The study was approved by the higher degrees research and ethics committee (HDREC) of Makerere University College of Health Sciences and finally by the Uganda National Council for Science and Technology (UNCST). Permission was sought from the local government in the districts of study and school authorities. Consent from the parents/guardians of the girls as well as assent from the girls were obtained.

### Study site

The study was conducted within two neighbouring districts of Western Uganda, Ibanda (vaccinated district) and Mbarara (unvaccinated) between October 2011- February 2012. Ibanda district covers an area of 967 square kilometres (373 sq mi) with one county and seven sub counties. It has an estimated population of 242,800 people. The vaccination exercise that was carried out was school based and targeted pupils form primary level 4 at an assumed age of 9 years. Mbarara district covers an area of 1,846.4 square kilometres (712.9 sq mi) with 4 counties and 19 sub counties. It has an estimated total population is about 457,800 people. At the time of the study, Ibanda had 180 primary schools with approximately 23,695 pupils out of which 6,770 (28.5%) pupils had been eligible for HPV-16/18 vaccination in 2010 by the PATH (Program for Appropriate Technology in Health) demonstration project. Mbarara district had 402 primary schools with about 50,307 pupils out which about 11,053 (21.9%) would have been eligible for vaccination in 2010 had the program covered Mbarara district.

### Selection of sub-counties

One sub-county from each district was purposively selected. The selection was based on the population of girls in primary level 5 being at least 500 girls in the sub-county. In addition, the sub-county had to be accessible to the main road so that blood samples could easily be delivered daily to the laboratory for processing.

### Selection of schools and girls in the vaccinated district

There were 29 schools in the selected sub-county of Ibanda out of which 15(51%) schools participated in the study. The schools were listed according to the number of eligible girls in descending order. Letters introducing the study and requesting for permission to carry out the study in the district schools were sent out to the district leaders. After receiving permission from the district leadership, phone contacts were made to the head teachers briefly explaining the study. Community mobilisers then visited all the schools within each sub county to deliver letters introducing the study and requesting for permission from the head teachers to have their schools participate in the study. A convenient day for the study team to visit the school was agreed upon. The mobilisers carried HPV vaccination registers from the district to verify the vaccination status of the girls who had been vaccinated in 2010. The head teachers were requested to invite the parents/guardians of the girls who had complete vaccine doses according to the register. On the day of the school visit, trained research assistants and the principal investigator (PI) explained the study to the girls and their parents/guardians in separate sessions. The explanations included the objectives of the study and procedures which involved answering a questionnaire and providing a blood sample. After the explanations and responding to questions, written informed consent from parents/guardians (guardians above 18 years) was obtained. In addition, a written assent from the girls whose parents had consented was also obtained. All girls who were fully vaccinated in 2010 and attending schools from within the selected sub-counties were eligible to participate. The girls were consecutively recruited until the desired sample size was realised.

### Selection of schools and girls in the unvaccinated district

Girls were eligible to participate if they were in the same class as the vaccinated ones and not repeaters of the same class. There were 34 schools in the selected sub-county of Mbarara out of which 17(50%) schools were involved in the study. The selection of the schools and visit procedures were similar to those employed in the vaccinated district. However, since the district had not participated in the vaccination program, there were no vaccine registers. Inquiry with the class teachers was made if any of the girls had previously been in the vaccinated district. A school register was used in addition to the information from the girls to verify the class level.

### Study procedures

In the vaccinated district, girls who were fully vaccinated (received 3 doses) in 2010 with assent and consent participated in the study. In the unvaccinated district only girls who would have been eligible to HPV vaccination at the same time as in the vaccinated district with assent and consent participated in the study. All the girls both vaccinated and unvaccinated answered an interviewer administered questionnaire that included sections on socio-demographic characteristics, vaccination status, general medical history and sexual history. The age was self reported by the girls and recorded as actual age at the time of the survey. Sexual activity was defined as having penetrative vaginal or anal sexual intercourse with the opposite sex. A blood sample was collected from both the vaccinated and the unvaccinated girls. At all times the blood sample was collected under aseptic conditions.

### Blood sample collection

The study laboratory technician, nurses and PI carried out the blood draws from the girls. In a sitting position, the girl’s non dominant hand was identified. Following identification of the vein in the cubital fossa, the area was cleaned with 70% alcohol swab. Two-three millilitres (*mls*) of blood were aseptically collected from the cubital vein into a labelled *SST*™ *tube*.

The vials of blood were put in transport boxes that had ice boxes and transported to Mbarara University Epicenter laboratory within 2–8 hours of collection. In the laboratory, the vials of blood were centrifuged for 15 minutes at 1000xg immediately on arrival. One millilitre of serum was aliquoted into pre-labelled 2 ml screw capped eppendorf tubes and temporarily stored at -80°C in Mbarara University Epicenter laboratory. Serum samples were then transported on dry ice to the Molecular laboratory at the Makerere University College of Health Sciences and kept at -80°C until shipping on dry ice to Germany Cancer Research Center (DKFZ) in Heidelberg.

### Serological evaluation for HPV antibodies

Antibodies to the L1 proteins of 8 hrHPV types (16, 18, 31, 33, 35, 45, 52, and 58) were analyzed using a multiplex serology method. This method is based on a Glutathione S-transferase capture immunosorbent assay combined with fluorescent-bead technology as described elsewhere [25-27].

### Procedure for estimating the HPV antibodies

Bead sets carrying different antigens were mixed and incubated with an equal volume of the serum dilutions (2 μl) in a 96 well plate. Human antibodies bound to their respective antigens were stained with a secondary biotinylated goat-anti human immunoglobulin IgG (Dianova, Hamburg, Germany) and the fluorescent reporter conjugate streptavidin-R-phycoerythrin. A Luminex100analyzer (Luminex100 IS™ by Luminex Corporation 12212 TechnologyBlvd. Austin, Texas 78727; http://www.luminexcorp.com) determined the internal bead colour specific for the respective antigen and reporter fluorescence of the beads, reflecting the quantity of antibodies recognizing the respective antigens. For each bead set the reporter fluorescence was expressed as median fluorescence intensity (MFI) of at least 100 beads per set per serum. Final antigen-specific MFI values were calculated by subtraction of Glutathione *S*-Transferase Fusion Proteins-tag (GST) and an individual bead background value. MFI were obtained for vaccine HPV-16/18 as well as non vaccine high risk HPV (hrHPV) 31, 33, 45, 52, 58. For proteins of HPV types (HPV 16, 18, 31, 33, 35, 45, 52, 58) cut-offs were calculated as mean plus 3 SD of sera from HPV negative controls. The MFI cut-offs were; 373, 200, 303, 200, 200, 200, 297, 275 for hrHPV types 16, 18, 31, 33, 35, 45, 52, 58 respectively.

### Sample size calculation

A sample size of 199 in each group would have 80% power to detect a difference in means of 4024.000 (the difference between a Vaccinated group mean, μ_1_ of 4029.000 and unvaccinated group mean, μ_2_, of 5.000) assuming that the common standard deviation is 14263.000 using a two group *t*-test with a 0.050 two-sided significance level [13].

We assumed 30% of the girls would either be absent or not have consent/assent; and that an additional 14% of the girls would have incomplete doses [28].

Therefore with the above assumptions, the target population would be at least 260 girls from Mbarara district (control district) and 288 girls from Ibanda district (Vaccinated).

### Data analysis

The data were analysed using *SPSS V20* (SPSS Hong Kong Ltd, Rm 1804, 18/F, Westlands Centre, Westlands Road, Quarry Bay, Hong Kong). At univariate analysis, categorical data were summarized using percentages while continuous variables were summarized by calculating the mean, standard deviation, median, range. Calculation of p values for mean comparison of age was done using Mann–Whitney *U* test while sexual activity between the two groups was compared using Pearson-chi square and Fishers exact test (p < 0.05significant).

MFI levels of antibodies to HPV-16/18 and other hrHPV types showed skewed distribution therefore the HPV antibody levels were transformed as Log_10_ (MFI levels for antibody concentration). We compared the mean of the MFI values between vaccinated and unvaccinated girls with the students *t* test. The mean of the MFI levels and corresponding 95% CI were calculated. Box plots present the distribution of the MFI values for HPV-16/18 antibodies levels among the vaccinated and unvaccinated girls at month 18 post vaccination. The median of the MFI values for the box plots was compared with the Mann–Whitney *U* test. In addition effect of age and sexual activity was compared to the median antibody levels among vaccinated girls and unvaccinated girls respectively. Significance level was set at p <0.05.

## Abbreviations

HPV: Human papilloma virus; MFI: Median fluorescent intensity; hrHPV: High risk human papilloma virus; UNCST: Uganda national council for science and technology; HDREC: Higher degrees research and ethics committee; DKFZ: Germany cancer research center; ELISA: Enzyme-linked Immunosorbent Assay; PI: Principal investigator; WHO: World Health Organisation.

## Competing interests

The authors declare that they have no competing interests.

## Authors’ contributions

NM, CB, PBN and FMM contributed towards the proposal development, data collection, interpretation of results, and/or writing and revising the manuscript and final decision to submit the manuscript. All authors read and approved the final manuscript.
